# The ER Stress Inducer l-Azetidine-2-Carboxylic Acid Elevates the Levels of Phospho-eIF2α and of LC3-II in a Ca^2+^-Dependent Manner

**DOI:** 10.3390/cells7120239

**Published:** 2018-11-30

**Authors:** Gemma Roest, Evelien Hesemans, Kirsten Welkenhuyzen, Tomas Luyten, Nikolai Engedal, Geert Bultynck, Jan B. Parys

**Affiliations:** 1 Laboratory for Molecular and Cellular Signaling, Department of Cellular and Molecular Medicine & Leuven Kanker Instituut, KU Leuven, Campus Gasthuisberg O/N-1 B-802, Herestraat 49, BE-3000 Leuven, Belgium; gemma.roest@kuleuven.be (G.R.); hesemans.evelien@kuleuven.be (E.H.); kirsten.welkenhuyzen@kuleuven.be (K.W.); tomas.luyten@kuleuven.be (T.L.); geert.bultynck@kuleuven.be (G.B.); 2 Centre for Molecular Medicine Norway, Nordic EMBL Partnership for Molecular Medicine, University of Oslo, P.O. Box 1137 Blindern, N-0318 Oslo, Norway; nikolai.engedal@ncmm.uio.no

**Keywords:** autophagy, ER stress, UPR, PERK, Ca^2+^, l-azetidine-2-carboxylic acid

## Abstract

Accumulation of misfolded proteins in the endoplasmic reticulum (ER) activates the unfolded protein response (UPR) to reduce protein load and restore homeostasis, including via induction of autophagy. We used the proline analogue l-azetidine-2-carboxylic acid (AZC) to induce ER stress, and assessed its effect on autophagy and Ca^2+^ homeostasis. Treatment with 5 mM AZC did not induce poly adenosine diphosphate ribose polymerase (PARP) cleavage while levels of binding immunoglobulin protein (BiP) and phosphorylated eukaryotic translation initiation factor 2α (eIF2α) increased and those of activating transcription factor 6 (ATF6) decreased, indicating activation of the protein kinase RNA-like ER kinase (PERK) and the ATF6 arms of the UPR but not of apoptosis. AZC treatment in combination with bafilomycin A1 (Baf A1) led to elevated levels of the lipidated form of the autophagy marker microtubule-associated protein light chain 3 (LC3), pointing to activation of autophagy. Using the specific PERK inhibitor AMG PERK 44, we could deduce that activation of the PERK branch is required for the AZC-induced lipidation of LC3. Moreover, both the levels of phospho-eIF2α and of lipidated LC3 were strongly reduced when cells were co-treated with the intracellular Ca^2+^ chelator 1,2-bis(*O*-aminophenoxy)ethane-*N*,*N*,*N′*,*N′*-tetraaceticacid tetra(acetoxy-methyl) ester (BAPTA-AM) but not when co-treated with the Na^+^/K^+^ ATPase inhibitor ouabain, suggesting an essential role of Ca^2+^ in AZC-induced activation of the PERK arm of the UPR and LC3 lipidation. Finally, AZC did not trigger Ca^2+^ release from the ER though appeared to decrease the cytosolic Ca^2+^ rise induced by thapsigargin while also decreasing the time constant for Ca^2+^ clearance. The ER Ca^2+^ store content and mitochondrial Ca^2+^ uptake however remained unaffected.

## 1. Introduction

Endoplasmic reticulum (ER) stress is a particular form of cellular stress that occurs when the ER machinery is overwhelmed by the amount of unfolded or misfolded proteins [1]. To restore ER homeostasis, a complex program is engaged that is called the unfolded protein response (UPR), and which consists of three arms, each depending on the activation of a distinct ER stress sensor, i.e., inositol-requiring enzyme 1 (IRE1), activating transcription factor (ATF) 6, and protein kinase RNA-like ER kinase (PERK). PERK activation leads to eukaryotic translation initiator factor 2α (eIF2α) phosphorylation and reduction of general protein translation, while IRE1 activation initiates mRNA degradation. Additionally, translocation of proteins into the ER is inhibited and macroautophagy (further called autophagy) is induced to eliminate damaged ER and to remove abnormal protein aggregates [2]. Furthermore, transcription factors dependent on each of the three arms of the response trigger the expression of a large variety of genes encoding proteins associated with ER-associated degradation, ER protein import, protein folding, lipid synthesis (needed for ER membrane expansion), as well as pro-survival genes including genes related to autophagy. The latter process largely depends on the PERK arm of the UPR that via eIF2α phosphorylation leads to the selective translation of ATF4 [2]. If the ER stress condition cannot be resolved, cell demise by apoptosis is eventually triggered [3].

Autophagy is an important, evolutionary conserved pro-survival process that promotes cellular homeostasis. Long-lived proteins and dysfunctional organelles are thereby engulfed in newly formed double-membrane vesicles called autophagosomes, which eventually will undergo fusion with lysosomes. In the resulting autolysosomes, the autophagosomes and their cargo are degraded and the components recycled [4].

A basal level of autophagy is always needed for the cell, but when cells undergo stress the autophagy pathway is upregulated in order to cope with the new situation and to regain cellular homeostasis. Apart from ER stress, cellular stress can also be the consequence of e.g., starvation conditions, the presence of intracellular pathogens or pharmacological compounds that induce autophagy [5]. It is a highly regulated process, which can be activated by adenosine monophosphate (AMP)-activated protein kinase or inhibited by the mechanistic target of rapamycin (mTOR) and that is further regulated by over 30 autophagy-related (ATG) proteins [6]. A long-standing question concerns the role of intracellular Ca^2+^ signaling in the regulation of the autophagic process and especially whether the role is stimulatory or inhibitory as evidence for both mechanisms have been proposed (recently reviewed in [7]).

ER stress itself is intimately linked to intracellular Ca^2+^ handling [8,9,10,11,12,13,14]. Treatment of cells with thapsigargin (TG) or cyclopiazonic acid, inhibitors of the sarco/endoplasmic Ca^2+^ ATPase (SERCA), leads to ER Ca^2+^ depletion and ER stress [15,16,17,18]. This is due to the fact that many ER chaperones play a dual role by not only participating in protein folding and maturation but also by binding the Ca^2+^ ions in the lumen of the ER [14].

Previous work by our own group had shown that intracellular Ca^2+^ was required for autophagic flux induced by either nutrient starvation [19], rapamycin treatment [20], or resveratrol treatment [21]. This conclusion was supported by the fact that incubation with the intracellular Ca^2+^ chelator 1,2-bis(*O*-aminophenoxy)ethane-*N*,*N*,*N*′,*N*′-tetraacetic acid tetra(acetoxymethyl) ester (BAPTA-AM), the inositol 1,4,5-trisphosphate receptor (IP_3_R) inhibitor Xestospongin B and/or IP_3_R knockout all inhibited autophagy induction. Moreover, these results are fully in line with those obtained by other groups [22,23,24,25,26,27,28]. However, at the best of our knowledge the Ca^2+^ sensitivity of autophagy occurring after ER stress as part of the UPR has never been tested, and it was the aim of this study to investigate this point.

**Figure 1:**
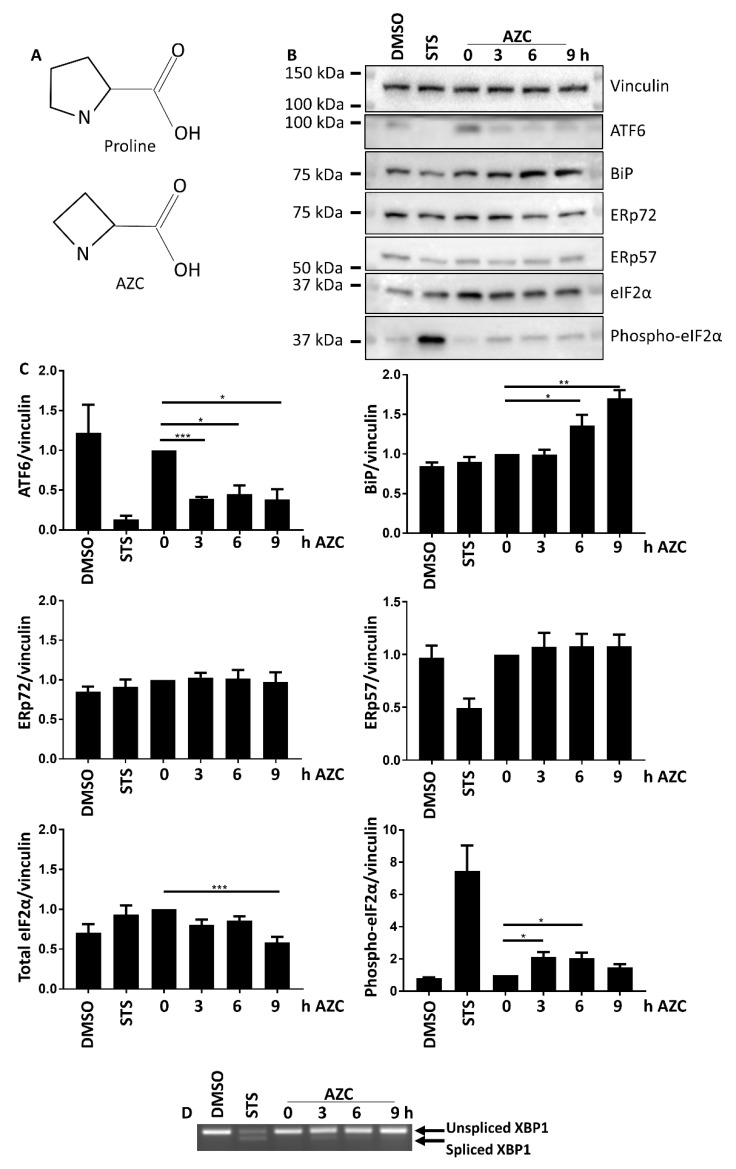
(**A**) l-azetidine-2-carboxylic acid (AZC) is an analog of proline. (**B**) AZC induces cleavage of ATF6 and upregulates BiP expression and phospho-eIF2α levels, but does not affect ERp57 or ERp72 expression. Cells were treated for 6 h with DMSO or 1 μM staurosporine (STS) as control, or for 0–9 h with 5 mM AZC, and during the last 4 h 100 nM Baf A1 was added. A representative blot for six independent experiments each performed in duplicate showing protein levels of ER stress markers ATF6, BiP, ERp72, ERp57, total eIF2α, and phospho-eIF2α. Vinculin was used as loading control. (**C**) Quantification of ER stress markers in B shown as mean ± SEM relative to 0 h AZC. * *p* < 0.05, ** *p* < 0.01, *** *p* < 0.001. (**D**) Representative RT-PCR gel of unspliced and spliced XBP1 mRNA levels of three independent experiments each performed in duplicate.

As for obvious reasons it was not appropriate to induce ER stress via a mechanism that by itself modified intracellular Ca^2+^, we could not use TG. Moreover, since autophagy and apoptosis influence each other [29,30,31] and apoptosis can occur subsequently to Ca^2+^ release by the IP_3_R and mitochondrial Ca^2+^ overload [32,33,34,35,36], we also had to avoid ER stress inducers that led to rapid apoptosis of the cells. We therefore focused on l-azetidine-2-carboxylic acid (AZC), a proline analog (Figure 1A) known to induce protein misfolding and aggregation and subsequent ER stress [37,38,39,40,41].

Our results indicate that Ca^2+^ plays an important role in the development of both the UPR and autophagy upon AZC treatment.

## 2. Materials and Methods

### 2.1. Cell Culture

HeLa cells were cultured at 37 °C and 5% CO_2_ in Dulbecco’s Modified Eagle Medium supplemented with 10% heat-inactivated fetal bovine serum (FBS), GlutaMAX (Gibco/Invitrogen, Merelbeke, Belgium; # 35050) and penicillin and streptomycin (Gibco/Invitrogen; # 15070-063), as described before [19,20,21]. Cells were washed with phosphate-buffered saline (PBS) and supplied with fresh medium two hours before the start of each experiment. The cell line has been authenticated using autosomal Short Tandem Repeat profiling performed by the University of Arizona Genetics Core and fully matched the DNA fingerprint present in the reference database.

### 2.2. Reagents and Antibodies

Reagents used were AZC (TCI Europe, Zwijndrecht, Belgium; # A1043 or Acros Organics, Geel, Belgium; # 105142500), bafilomycin A1 (Sanbio, Uden, The Netherlands; # 11038-500), ethylene glycol tetraacetic acid (Acros Organics; # 409910250), BAPTA-AM (Thermo Fisher Scientific, Waltham, MA, USA; # B6769), TG (Alomone labs, Jerusalem, Israel; # T-650), Fura-2 AM (Life Technologies, Carlsbad, CA, USA; # F1221), staurosporine (LC Labs, Woburn, MA, USA; # S9300), and AMG PERK 44 (Tocris, Abingdon, U.K.; # 5517).

Primary antibodies used were anti-ATF6 (Cell Signaling Technology, Leiden, The Netherlands; # 65880), anti-BiP (Cell Signaling Technologies; # 3183), anti-eIF2α (Cell Signaling Technology; # 9722), anti-phospho-eIF2α (Cell Signaling Technology; # 3398), anti-ERp57 (Cell Signaling Technology; # 2881), anti-ERp72 (Cell Signaling Technology; # 2798), anti-GAPDH (Sigma-Aldrich, St. Louis, MO, USA; # G8795), anti-IP_3_R (Rbt475 [42] recognizing all IP_3_R isoforms), anti-LC3 (Cell Signaling Technology; # 2775), anti-MCU (Sigma-Aldrich; # HPA016480), anti-PARP (Cell Signaling Technology; # 9532), anti-PMCA (Thermo Fisher Scientific; # MA3-914) recognizing all PMCA isoforms, anti-SERCA2B (Cell Signaling Technology; # 4435), and anti-vinculin (Sigma-Aldrich; # V-9131).

### 2.3. Sodium Dodecyl Sulfate (SDS) Polyacrylamide Gel Electrophoresis and Western Blotting

Cells were washed with PBS and lysed with lysis buffer (150 mM Hepes (pH 7.5), 150 mM NaCl, 100 mM NaF, 10 mM ethylene diamine tetraacetic acid (EDTA), 10 mM Na_4_P_2_O_7_, 1% Triton-X-100, 0.1% SDS, EDTA-free protease inhibitor (Thermo Fisher Scientific; # 88266), PhosSTOP phosphatase inhibitor (Sigma-Aldrich; # 04906837001)). Lysates were incubated on ice for 30 min and centrifuged for 5 min at 8000× *g*. Protein concentration of the supernatant was determined using a bicinchonic acid protein assay kit and bovine serum albumin standards (Thermo Fisher Scientific; # 23225). Proteins were then separated on 10–20% Tris-glycine gels (Thermo Fisher Scientific) using Tris-glycine running buffer (Thermo Fisher Scientific; # LC2675). Proteins were transferred to polyvinylidene difluoride membrane in running buffer (25 mM Trizma base, 192 mM glycine) containing 10% methanol and blocked in 5% non-fat milk in Tris-buffered saline (TBS) containing 0.1% Tween. Membranes were incubated with primary antibodies overnight at 4 °C, and horseradish peroxidase-conjugated secondary antibodies for 1 h at room temperature. Proteins were detected using Clarity Western Enhanced Chemiluminescence (ECL) Substrate (Biorad, Temse, Belgium; # 170-5061) or ECL Western Blotting Substrate (Thermo Fisher Scientific; 32106). Individual experiments were always performed in duplicate.

### 2.4. XBP1 Splicing

Cells were trypsinized and centrifuged for 5 min at 500× *g*. RNA was extracted using the High Pure RNA Isolation kit (Roche, Mannheim, Germany; # 11828665001) according to manufacturer’s protocol. cDNA was prepared using the High Capacity cDNA Reverse Transcription kit (Applied Biosystems, Brussels, Belgium; # 4368814) according to manufacturer’s protocol. XBP1 mRNA was amplified using GoTaq Green master mix (Promega, Leiden, The Netherlands; # M7112) and XBP1 specific primers (IDT, Leuven, Belgium) and separated on a 2.5% Ultrapure agarose (Invitrogen, # 16500-500) gel containing 0.005% EtBr (Invitrogen, # 15585-011). Individual experiments were always performed in duplicate.

### 2.5. Cell Death Assays

Cell death was assessed by PARP cleavage and by propidium iodide (PI) staining. PARP cleavage was assessed by Western blotting as described in Section 2.3. PI staining was performed essentially as previously described [43]. Briefly, cells were plated in 96-well plates and stained with 2.5 μg/mL PI (Thermo Fisher Scientific; # P3566). Cells were treated with 0–25 mM AZC, and the PI fluorescence was monitored for a total of 72 h in an IncuCyte Zoom (Essen Bioscience, Welwyn Garden City, UK) with a 4 h interval between scans. Individual experiments were always performed in triplicate.

### 2.6. Ca^2+^ Measurements at the Population Level

Cells were plated in 96-well plates and treated as indicated. During the last hour of treatment the cells were loaded for 30 min with 1.8 μM Fura-2 AM followed by 30 min de-esterification in modified Krebs solution (150 mM NaCl, 5.9 mM KCl, 1.2 mM MgCl_2_, 11.6 mM Hepes (pH 7.3), 11.5 mM glucose, 1.5 mM CaCl_2_). Fluorescence was measured on a FlexStation 3 microplate reader (Molecular Devices, Sunnyvale, CA, USA) by alternate excitation at 340 nm and 380 nm and recording emission at 510 nm. Compounds were added as indicated. Individual experiments were always performed in triplicate.

### 2.7. Single-Cell Ca^2+^ Measurements

ER and mitochondrial Ca^2+^ levels were measured with the genetically encoded Ca^2+^ indicators G-CEPIA1*er* and R-GECO1*mt* [44] kindly provided by Dr. M. Iino (The University of Tokyo, Tokyo, Japan). Cells were transfected with 300 ng G-CEPIA1*er* and 600 ng R-GECO1*mt* using X-treme Gene HP DNA (Roche; # 06366546001) according to the manufacturer’s protocol. After 48 h single-cell measurements were performed on a Zeiss Axio Observer Z1 Inverted Microscope equipped with a 20× air objective and a high-speed digital camera (Axiocam Hsm, Zeiss, Jena, Germany). Extracellular Ca^2+^ was chelated with 3 mM ethylene glycol tetraacetic acid (EGTA) and one minute later the indicated compound was added. Changes in G-CEPIA1*er* fluorescence were followed after excitation at 480 nm and measurement of emission at 520 nm. Changes in R-GECO1*mt* fluorescence were followed after excitation at 377 nm and measurement of emission at 466 nm. The traces were normalized to baseline fluorescence (F/F_0_) where the baseline was calculated as the average of the first six time points. At least 99 cells per condition were measured in the course of six individual experiments.

### 2.8. Statistics

Results are presented as mean ± standard error of the mean (SEM). Significance was tested using a one-way analysis of variance with Tukey post-hoc test. Results were considered significant when *p* < 0.05.

## 3. Results

### 3.1. AZC Upregulates the Levels of BiP and Phospho-eIF2α while Decreasing the Level of Full-Length ATF6

To analyze the induction of ER stress, HeLa cells were treated with 5 mM AZC for up to 9 h. The cell lysates were analyzed by Western blotting for the ER stress markers ATF6, binding immunoglobulin protein (BiP), ER protein (ERp) 72, ERp57, and total and phospho-eIF2α (Figure 1B,C). AZC treatment led to a 2.5-fold decrease in full-length ATF6 (already significantly decreased after 3 h), a 1.5-fold increase of BiP protein levels (already significantly increased after 6 h), and a 2-fold increase in the levels of phospho-eIF2α (already significantly increased after 3 h). The total level of eIF2α only decreased after treatment with AZC for 9 h, while ERp57 or ERp72 protein levels did not change in response to AZC treatment. To evaluate the activation of the IRE1α arm of the UPR, we performed an X-box protein (XBP) 1 splicing assay. AZC treatment did not significantly induce XBP1 splicing (Figure 1D). Staurosporine (STS, 1 µM) was used as a positive control for activation of ER stress [45] and cell death [46,47,48]. STS treatment for 6 h resulted in a 10-fold reduction in the level of full-length ATF6, a two-fold downregulation of ERp57 protein levels, an approximately nine-fold increase in the levels of phospho-eIF2α and a substantial production of spliced XBP1, but did not modify BiP levels.

Taken together, these results indicate that a 6 h treatment with AZC activates both the PERK and the ATF6 arm of the UPR, resulting in upregulated BiP protein expression and increased phosphorylation of eIF2α.

### 3.2. AZC Does Not Induce Cell Death within 6 h of Treatment

**Figure 2:**
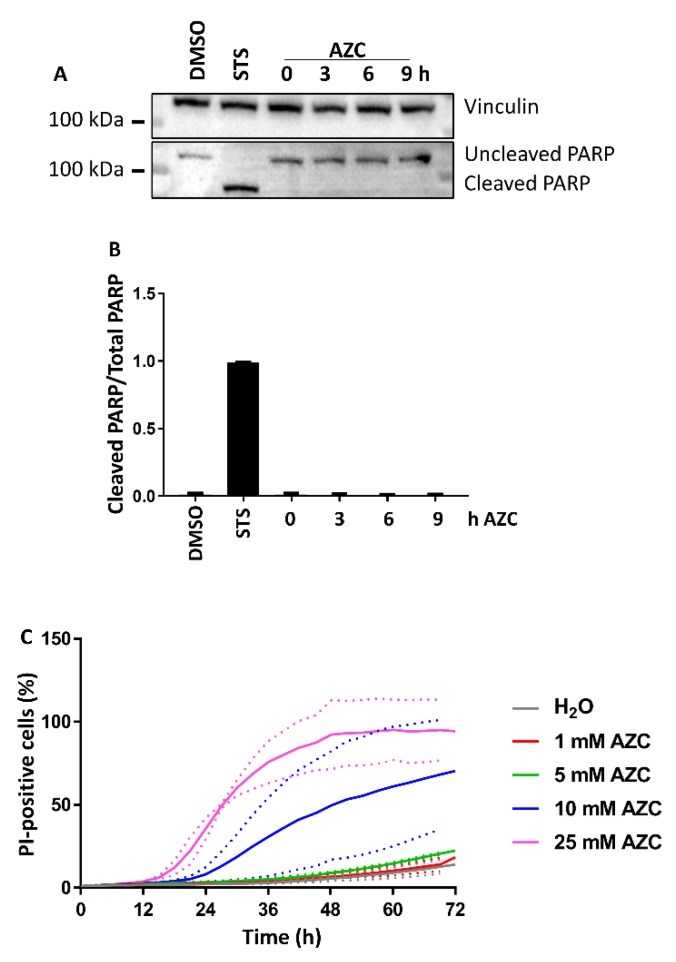
AZC does not induce cell death within 6 h of treatment. (**A**) A representative blot of six independent experiments each performed in duplicates showing protein levels of uncleaved and cleaved poly adenosine diphosphate ribose polymerase (PARP). Cells were treated for 6 h with DMSO or 1 μM staurosporine (STS) as control, or for 0–9 h with 5 mM AZC, and during the last 4 h 100 nM Baf A1 was added. Vinculin was used as loading control. (**B**) Quantification of PARP cleavage in (**A**) shown as mean ± SEM relative to 0 h AZC. (**C**) AZC induces cell death in a concentration-dependent manner only from 12 h of treatment. Cells were stained with 2.5 μg/mL propidium iodide (PI) and treated with 0–25 mM AZC for 72 h. Results are shown as average ± SEM (indicated by the dotted lines) of two experiments each performed in triplicate.

Because it was important to study ER stress and subsequent autophagy in conditions where cell death was not induced, we assessed the levels of cleaved poly adenosine diphosphate ribose polymerase (PARP) upon AZC treatment during the same time period. While STS led to clear PARP cleavage, we observed virtually no cleavage upon AZC treatment (Figure 2A). The quantification of these results is shown in Figure 2B. Additionally, we evaluated the cellular toxicity of various AZC concentrations as a function of time by monitoring the increase of PI-positive cells. These results indicated a dose-dependent increase of PI-positive cells with time starting only after at least 12 h of treatment with AZC, even at high AZC concentrations (10–25 mM). Moreover, even after 72 h, 5 mM AZC induced less than 10% cell death above the vehicle control condition.

From both the PARP-cleavage analysis and the PI staining, we can conclude that treatment with 5 mM AZC for 6 h did not lead to any significant cell death.

### 3.3. AZC Increases the Levels of Lipidated Autophagy Marker LC3

**Figure 3:**
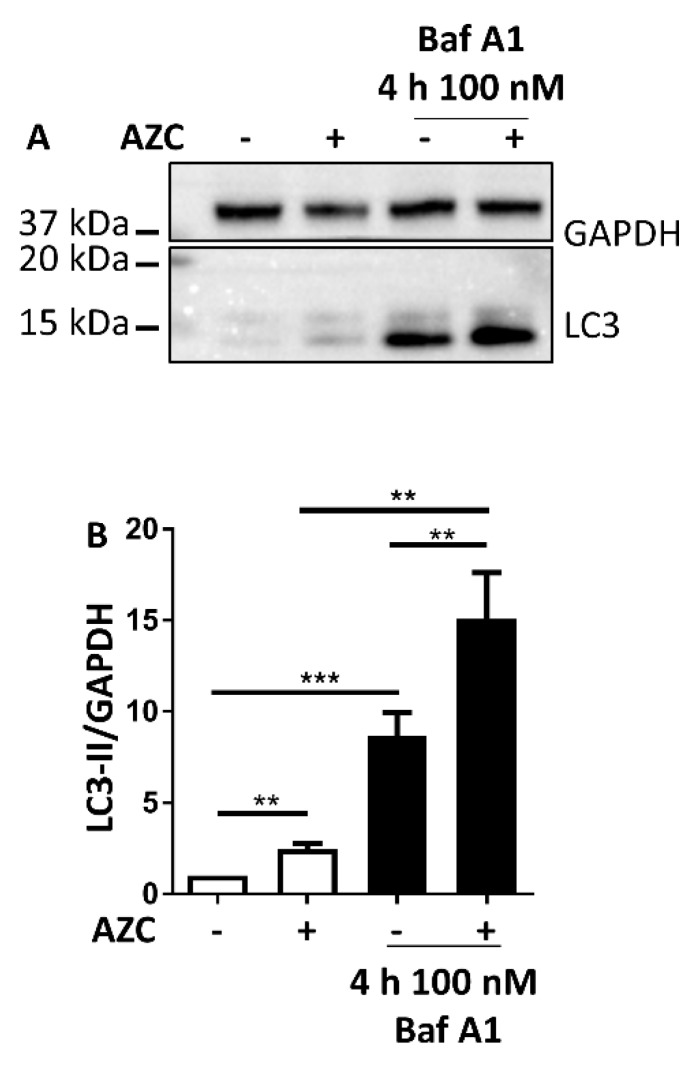
AZC induces autophagy. Cells were treated for 6 h with 5 mM AZC, and during the last 4 h 100 nM bafilomycin A1 (Baf A1) was added to inhibit autolysosomal degradation. (**A**) A representative blot of twelve independent experiments each performed in duplicate showing LC3-II protein levels. Glyceraldehyde 3-phosphate dehydrogenase (GAPDH) was used as loading control. (**B**) Quantification of LC3-II levels in (**A**) shown as mean ± SEM relative to untreated control. ** *p* < 0.01, *** *p* < 0.001.

To avoid interference of ER stress-induced cell death, we wanted to use conditions that allowed induction of the UPR and of autophagy in a time frame as short as possible. Based on the results presented in Figure 1 (activation of the UPR) and in Figure 2 (absence of cell death), we decided to select a 6 h incubation time with 5 mM AZC to assess the levels of the autophagy marker lipidated microtubule-associated protein light chain 3 (LC3-II). In order to evaluate autophagy induction upon AZC treatment, autolysosomal degradation was inhibited by addition of 100 nM bafilomycin A1 (Baf A1) during the last 4 h of AZC treatment. In the absence of Baf A1, AZC increased the levels of LC3-II 3-fold as assessed by Western blotting (Figure 3A). Co-treatment with Baf A1 increased the levels of LC3-II about 8- and 13-fold in control and AZC-treated cells, respectively. Figure 3B shows the quantification from twelve independent experiments.

The larger increase of LC3-II levels in AZC-treated cells in the presence of Baf A1 as compared to the control conditions suggests that the upregulation of LC3-II levels does not depend on a block of autophagosome-lysosome fusion and/or of lysosomal degradative activity but is related to the induction of autophagy.

### 3.4. AZC Upregulates the Levels of LC3-II Subsequently to Activation of the PERK Pathway

**Figure 4:**
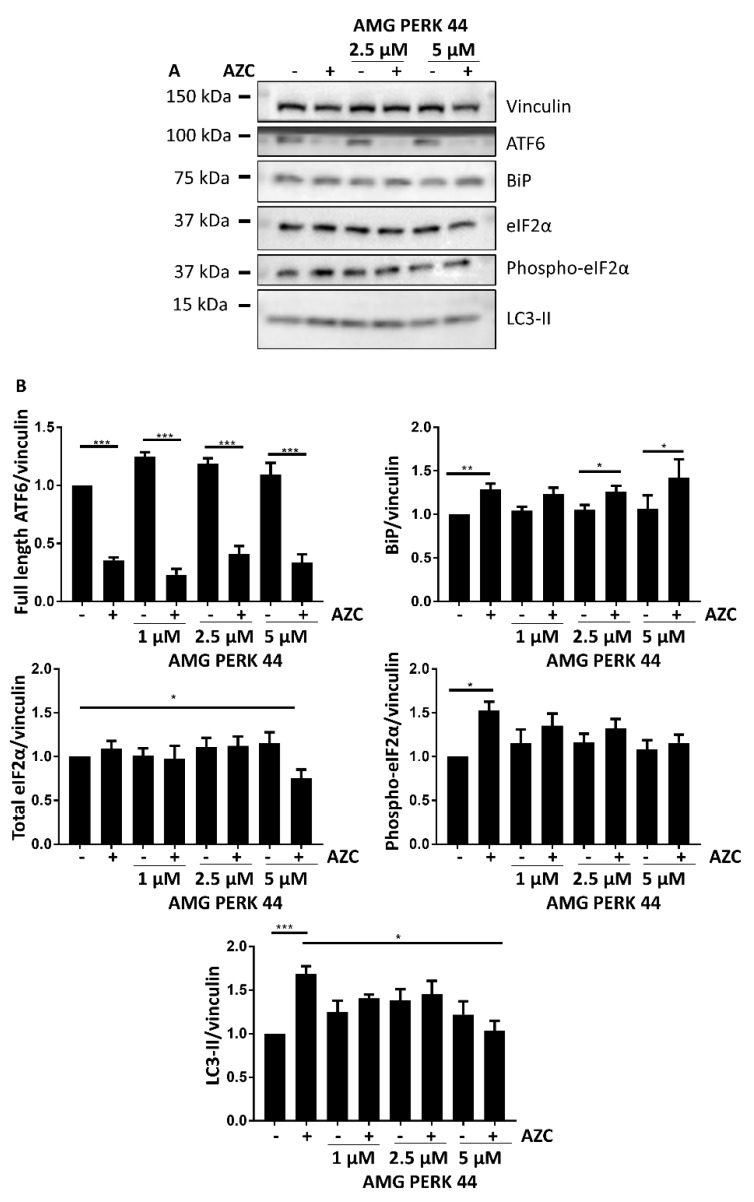
AZC elevates LC3-II levels in a PERK-dependent manner. Cells were treated for 6 h with 5 mM AZC in absence or presence of 1 μM, 2.5 μM, or 5 μM of the PERK inhibitor AMG PERK 44, and during the last 4 h 100 nM Baf A1 was added. (**A**) A representative blot for six independent experiments each performed in duplicate in absence or presence of AMG PERK 44 (2.5 and 5 μM) showing protein levels of ER stress markers ATF6, BiP, total eIF2α, and phospho-eIF2α and of autophagy marker LC3-II. Vinculin was used as loading control. (**B**) Quantification of ER stress and autophagy markers in (**A**) as well as of independent experiments performed in absence or presence of AMG PERK 44 (1–5 μM), shown as mean ± SEM relative to untreated control. * *p* < 0.05, ** *p* < 0.01, *** *p* < 0.001.

To investigate the relation between the UPR and the increased LC3-II levels after application of AZC, we used the PERK-selective inhibitor AMG PERK 44 [49]. AMG PERK 44 (1–5 µM) did neither affect ATF6 cleavage nor BiP induction (Figure 4A,B). Not only the AZC-induced increase in phospho-eIF2α was abolished, as was anticipated, but also the AZC-induced increase in LC3-II in spite of the fact that the ATF6 pathway remained active.

These results indicate that activation of the PERK pathway is a prerequisite for the occurrence of the AZC-induced increase in LC3-II levels, and that the ATF6 branch of the UPR is not sufficient for obtaining this effect.

### 3.5. AZC Upregulates the Levels of LC3-II in a Ca^2+^-Dependent Manner

**Figure 5:**
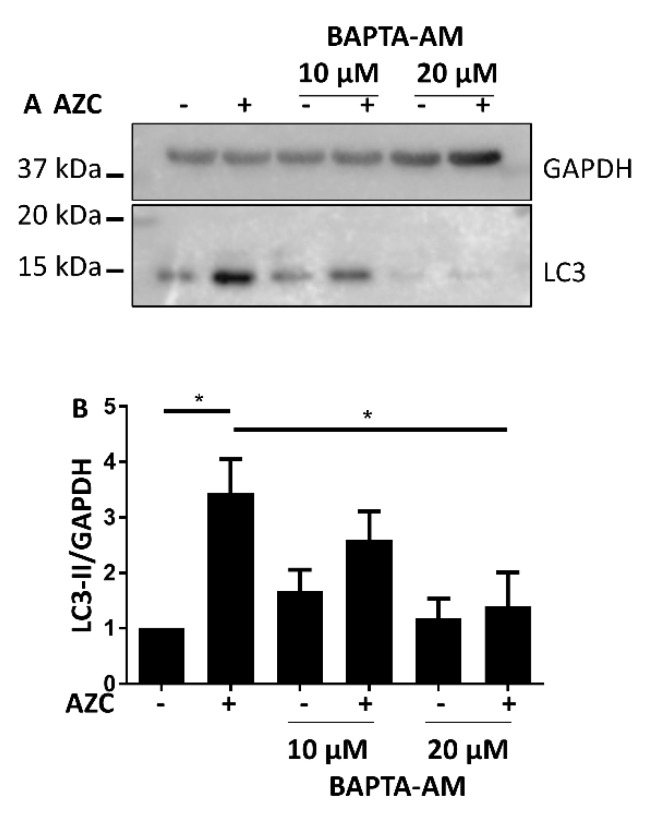
AZC elevates LC3-II levels in a Ca^2+^-dependent manner. Cells were treated for 6 h with 5 mM AZC in absence or presence of 10 μM or 20 μM of the intracellular Ca^2+^ chelator BAPTA-AM, and during the last 4 h 100 nM Baf A1 was added. (**A**) A representative blot of six independent experiments each performed in duplicate, assessing protein levels of the autophagy marker LC3-II. Glyceraldehyde 3-phosphate dehydrogenase (GAPDH) was used as loading control. (**B**) Quantification of LC3-II levels in (**A**) shown as mean ± SEM relative to untreated control. * *p* < 0.05.

To investigate the role of Ca^2+^ in the elevation of LC3-II levels triggered by AZC, we co-treated the cells with increasing concentrations of the intracellular Ca^2+^ chelator BAPTA-AM. This led to a dose-dependent reduction of the AZC-induced increase in LC3-II (Figure 5A). Figure 5B shows the quantification from six individual experiments.

These results strongly suggest that the AZC-induced increase in LC3-II requires intracellular Ca^2+^.

### 3.6. AZC-Induced Elevation of Phospho-eIF2α Levels Is Ca^2+^ Dependent

**Figure 6:**
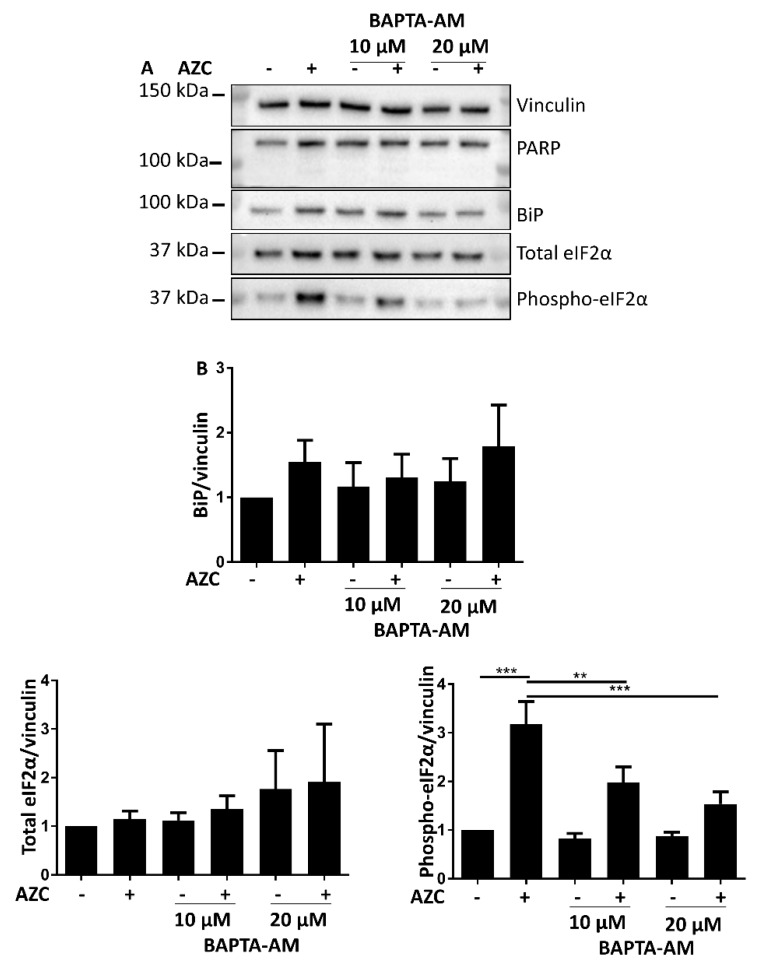
AZC-induced elevation of phospho-eIF2α levels is Ca^2+^ dependent. Cells were treated for 6 h with 5 mM AZC in absence or presence of 10 μM or 20 μM of the intracellular Ca^2+^ chelator BAPTA-AM and during the last 4 h 100 nM Baf A1 was added. (**A**) Representative blot of six independent experiments each performed in duplicate showing protein levels of the cell death marker PARP, and of ER stress markers BiP, total eIF2α and phospho-eIF2α. Vinculin was used as loading control. (**B**) Quantification of ER stress markers in (**A**) shown as mean ± SEM relative to untreated control. ** *p* < 0.01, *** *p* < 0.001.

The link between ER stress induction and autophagy involves several steps. In order to identify which steps are sensitive to Ca^2+^, we assessed the effect of BAPTA-AM on the AZC-induced upregulation of BiP expression levels, and on the levels of phospho-eIF2α. While there was no effect of BAPTA-AM treatment on BiP levels, we observed a significant reduction of AZC-induced elevation of phospho-eIF2α upon co-treatment with BAPTA-AM (Figure 6A,B). Additionally, we verified that BAPTA-AM treatment did not result in PARP cleavage (Figure 6A).

These results indicate that levels of phospho-eIF2α induced by AZC is dependent on Ca^2+^, and may thus constitute an upstream event to the sensitivity of the subsequent autophagic process to Ca^2+^.

### 3.7. Effects of BAPTA-AM Treatment Are Not Related to Na^+^/K^+^ ATPase Inhibition

**Figure 7:**
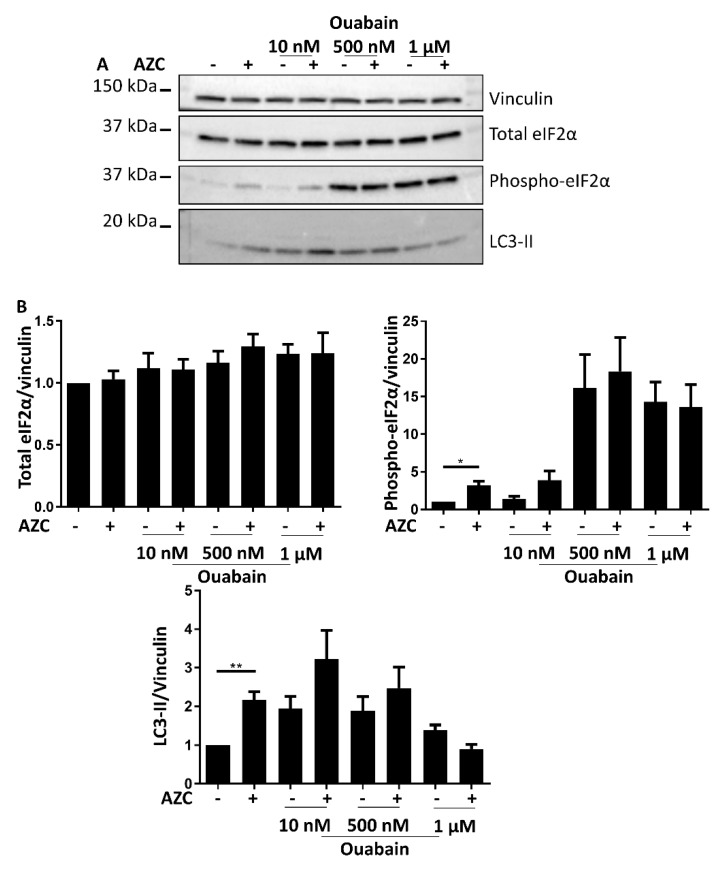
Ouabain affects ER stress, apoptosis and autophagy markers differently than BAPTA-AM. Cells were treated for 6 h with 5 mM AZC in absence or presence of 10 nM, 500 nM, or 1 μM of the Na^+^/K^+^ ATPase inhibitor ouabain, and during the last 4 h 100 nM Baf A1 was added. (**A**) A representative blot of eight independent experiments each performed in duplicate showing levels of total eIF2α and phospho-eIF2α, and of LC3-II. Vinculin was used as loading control. (**B**) Quantification of protein levels in (**A**) shown as mean ± SEM relative to untreated control. * *p* < 0.05, ** *p* < 0.01.

Recently, it was shown that BAPTA-AM can also affect Na^+^/K^+^ ATPase activity [50]. To discriminate the Ca^2+^-chelating role of BAPTA-AM from its inhibitory effect on the Na^+^/K^+^ ATPase, we combined AZC treatment with various concentrations of the specific Na^+^/K^+^ ATPase inhibitor ouabain and assessed the levels of total and phospho-eIF2α and the levels of LC3-II. In contrast to the effects observed with BAPTA-AM, increasing ouabain concentrations resulted in strongly increased levels of phospho-eIF2α, while LC3-II levels generally increased up to 500 nM ouabain followed by a decrease at the highest used concentration of ouabain (Figure 7A,B). Moreover, these effects occurred independently of the absence or presence of AZC.

These results are therefore distinct from those obtained with BAPTA-AM (Figure 5 and Figure 6) and thus support a role for intracellular Ca^2+^ in AZC-induced ER stress and autophagy.

### 3.8. Pretreatment with AZC Reduces the Cytosolic Amount of Ca^2+^ after ER Store Release

**Figure 8:**
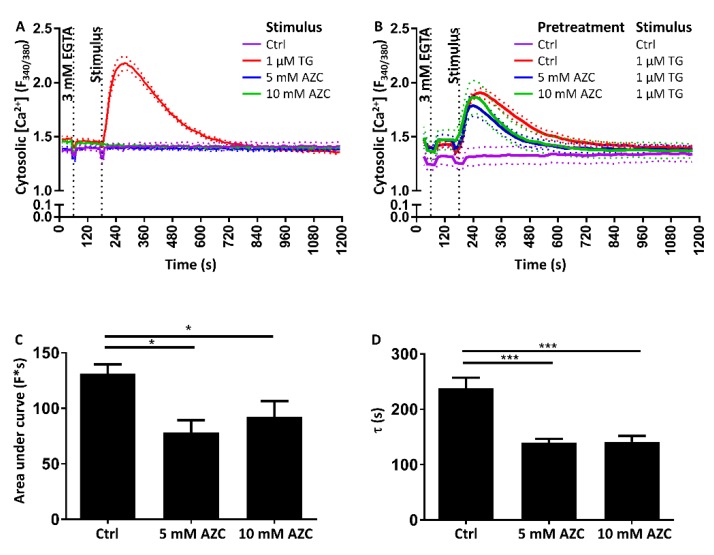
AZC does not acutely affect cytosolic Ca^2+^ but pretreatment with AZC reduces the Ca^2+^ amount detected in the cytosol after ER Ca^2+^ store release evoked by thapsigargin (TG). Results are shown as mean ± SEM (indicated by the dotted lines) of three independent experiments each performed in duplicate. (**A**) Cells were loaded with 1.8 μM Fura-2 AM for 30 min, followed by 30 min of deesterification. Fura-2 AM fluorescence was monitored using a FlexStation 3 microplate reader. Extracellular Ca^2+^ was chelated with 3 mM EGTA and 120 s later AZC or 1 μM TG (as a positive control) was added as indicated. (**B**) Cells were pretreated for 6 h with 5 mM or 10 mM AZC. During the last hour of treatment, cells were loaded with 1.8 μM Fura-2 AM for 30 min, followed by 30 min of deesterification. Extracellular Ca^2+^ was chelated with 3 mM EGTA and 120 s later 1 μM TG or Krebs solution (vehicle) was added as indicated. (**C**) Quantification of the area under the curve of the Ca^2+^ traces in (**B**) * *p* < 0.05. (**D**) Time constant τ for the decline phase of the Ca^2+^ traces in (**B**) *** *p* < 0.001.

To determine the relation between AZC treatment and intracellular Ca^2+^ handling, we investigated whether AZC treatment directly impacts the Ca^2+^ stores. We therefore loaded the cells with the cytosolic Ca^2+^ indicator Fura-2 AM and followed its fluorescence on a FlexStation 3 microplate reader. To prevent influx of Ca^2+^ in the cell, the extracellular Ca^2+^ was first chelated by addition of 3 mM EGTA, and the SERCA inhibitor TG was subsequently used to uncover Ca^2+^ release from the ER. While TG led to an increase in cytosolic [Ca^2+^], acute application of either 5 mM or 10 mM AZC did not provoke a rise in cytosolic [Ca^2+^] (Figure 8A). However, when cells were pre-treated for 6 h with 5 mM or 10 mM AZC and TG-induced [Ca^2+^] elevations were monitored, we noticed a lower rise of cytosolic Ca^2+^ in the pre-treated samples as compared to the control samples (Figure 8B). This was reflected by a ~35% lower area under the curve (Figure 8C). Additionally, the clearance of Ca^2+^ from the cytosol appeared faster after AZC pretreatment, as indicated by a ~42% lower τ value for the decline phase of the Fura-2 AM signal (Figure 8D).

In short, AZC does not have an acute effect on intracellular Ca^2+^, though a prolonged incubation with the compound appears to modulate intracellular Ca^2+^ signaling in a complex way.

### 3.9. AZC Does Not Affect the ER Ca^2+^ Store Content or the ER-Mitochondrial Ca^2+^ Transfer

**Figure 9:**
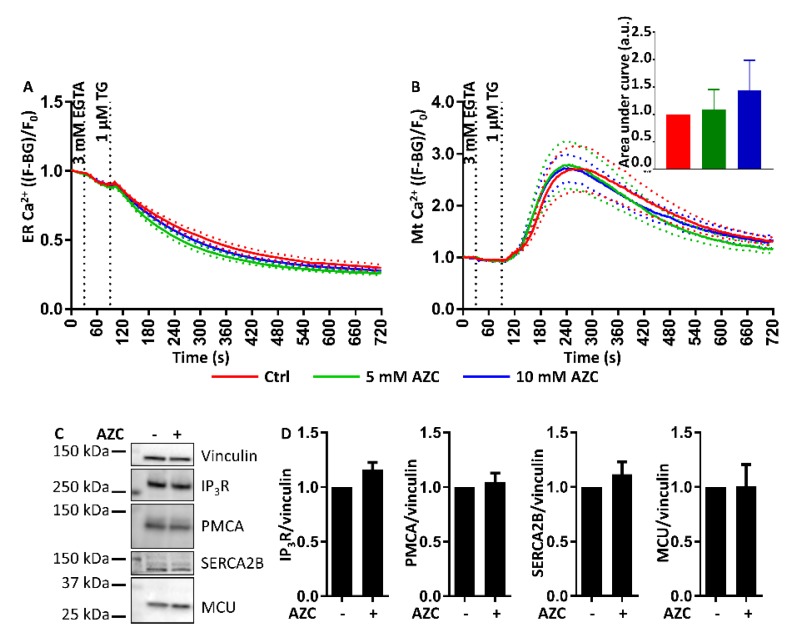
AZC does not affect the ER store content or ER-mitochondrial Ca^2+^ transfer. Cells were transfected with the Ca^2+^ sensors G-CEPIA1*er* and R-GECO1*mt*. At 48 h after transfection, cells were pretreated with 5 mM or 10 mM AZC for 6 h. Extracellular Ca^2+^ was chelated with 3 mM EGTA and 60 s later ER Ca^2+^ release was uncovered with 1 μM thapsigargin (TG) as indicated. At least 99 cells in six independent experiments were measured and results are shown as mean ± SEM (indicated by the dotted lines). (**A**) AZC pretreatment did not change the properties of the ER Ca^2+^ store as detected by G-CEPIA1*er*. (**B**) AZC pretreatment did not affect uptake by the mitochondria of ER-released Ca^2+^ as detected by R-GECO1*mt.* The insert shows the quantification of the area under the curve of traces showing Ca^2+^ uptake by the mitochondria. (**C**) AZC treatment does not affect the expression levels of the IP_3_Rs, the plasma membrane Ca^2+^ ATPases (PMCA), SERCA2B or the mitochondrial Ca^2+^ uniporter (MCU). A representative blot of eight independent experiments each performed in duplicate is shown. Vinculin was used as loading control. (**D**) Quantification of protein levels in (**C**) shown as mean ± SEM relative to untreated control.

Because we observed a lower rise in cytosolic [Ca^2+^] upon application of TG after pretreatment with AZC, we hypothesized that AZC may lower the ER Ca^2+^ store content, or that Ca^2+^ transfer to the mitochondria may have been increased. In order to investigate this, we co-transfected cells with both G-CEPIA1*er* and R-GECO1*mt* to simultaneously detect ER Ca^2+^ and mitochondrial Ca^2+^, and treated the cells for 6 h with 5 mM or 10 mM AZC. EGTA was then applied and Ca^2+^ released from the ER with TG. Surprisingly, we did not detect differences in the ER Ca^2+^-leak rates between cells pretreated with either 5 mM or 10 mM AZC and untreated control cells (Figure 9A). Furthermore, the amount of Ca^2+^ transferred into the mitochondria after TG application was also very similar between AZC-treated cells and untreated cells (Figure 9B). Finally, the difference in the TG-triggered cytosolic [Ca^2+^] rise between AZC-treated and untreated cells could also not be linked to a change in expression levels of any of the major Ca^2+^-transport proteins, such as IP_3_Rs, PMCAs, SERCA2B, or the mitochondrial Ca^2+^ uniporter (MCU) (Figure 9C,D).

Taken together, long-term treatment of cells with AZC (6 h) seems to modify cellular Ca^2+^ handling, though without directly affecting the ER Ca^2+^ leak, ER-mitochondrial Ca^2+^ transfer or the expression of IP_3_Rs, PMCAs, SERCA2B, and the MCU.

## 4. Discussion

One of the main functions of the ER is the proper folding of nascent proteins. To ensure this, chaperones assist and correct the process when necessary [51]. Stress conditions including oxidative stress or high temperatures can disturb the folding and cause an accumulation of misfolded proteins in the ER and subsequently ER stress. Activation of the UPR can resolve this situation but failure to do so may result in accumulation of protein aggregates and eventually cell death [52]. Prolonged ER stress may also be involved in neurodegenerative diseases including Alzheimer’s disease, type 2 diabetes, and cancer [53].

Classic chemical ER stress inducers include dithiothreitol, tunicamycin, and TG. In our preliminary experiments however, these compounds also showed induction of apoptosis or they displayed an intrinsic effect on Ca^2+^ homeostasis. Therefore, we turned to AZC. This proline analogue contains a four-membered carbon ring in contrast to the five-membered ring in proline (Figure 1A). Incorporation of AZC in proteins leads to an altered tertiary protein structure and accumulation of protein aggregates [37,38,54]. Our results indicate that this activates in HeLa cells the PERK and the ATF6 arms of the UPR but not the IRE1 arm (Figure 1). This is in line with the results of Qian et al. who in HEK293 cells found a dose- and time-dependent increase of phospho-eIF2α upon AZC treatment [39], and with Shang et al. who showed that significant XBP1 splicing requires very high concentrations of AZC [41]. The former correlated with a decrease in phosphorylation of ribosomal protein S6, indicating inhibition of the autophagy suppressor mTOR. This is in line with our results with Baf A1 (Figure 3), which suggest a role of AZC in the induction of autophagy. Moreover, using the specific PERK inhibitor AMG PERK 44, we can conclude that activation of the PERK arm of the UPR is absolutely needed for the subsequent increase in LC3-II levels by AZC. Finally, Nivon et al. showed that AZC treatment causes the formation of protein aggregates in HeLa cells and that this correlates with an NFκB-dependent increase in LC3-II levels [38].

Ca^2+^ plays an important role in both ER stress and autophagy [7,9,14]. In autophagy, depending on the exact conditions both inhibitory and stimulatory effects of Ca^2+^ were described [7,55]. To assess the role of Ca^2+^ in intracellular processes like autophagy, treatment with the intracellular Ca^2+^ chelator BAPTA-AM is the favored technique [7]. We therefore co-treated our cells with AZC and BAPTA-AM, upon which we observed a decrease of AZC-induced elevation of phospho-eIF2α as well as of LC3-II (Figure 5 and Figure 6). However, recently it was shown that various related Ca^2+^-binding molecules, including BAPTA-AM, can inhibit the Na^+^/K^+^ ATPase, independently of their Ca^2+^-binding affinity [50]. Although in the latter study, BAPTA-AM was a less potent Na^+^/K^+^ ATPase inhibitor than the other compounds tested, it readily accumulates in the cell and we therefore wanted to verify whether Na^+^/K^+^ ATPase inhibition by BAPTA-AM could affect the interpretation of our results. One way to discriminate between the Ca^2+^ chelating and the Na^+^/K^+^ ATPase inhibiting effect of BAPTA-AM, is to assess whether the effects provoked by BAPTA-AM are replicated by the well-known specific Na^+^/K^+^ ATPase inhibitor ouabain [56]. All human Na^+^/K^+^ ATPase isozymes are characterized by a K_d_ for ouabain between 13 and 37 nM [57]. In order to encompass various levels of Na^+^/K^+^ ATPase inhibition, we applied a range of concentrations of ouabain between 10 nM and 1 µM. However, at none of these concentrations did ouabain (Figure 7) mimic the effect of BAPTA-AM on AZC-induced elevation of phosphorylated eIF2α and LC3-II (Figure 5 and Figure 6). Therefore, we conclude that these effects represent a bona fide contribution of Ca^2+^ chelation rather than Na^+^/K^+^ ATPase inhibition and that intracellular Ca^2+^ is critical for both AZC-induced ER stress and elevation of LC3-II.

Interestingly, our data indicate an essential role of Ca^2+^ in AZC-induced elevation of phospho-eIF2α. Given that the PERK-arm of the UPR has been reported to be required for ER stress-mediated induction of LC3-II [58,59], this finding may form a link with the requirement for Ca^2+^ observed for the subsequent elevation of LC3-II levels. Whereas Ca^2+^ was recently shown to be required for non-UPR-related functions of PERK [60], our study indicates that Ca^2+^ is also required for efficient eIF2α phosphorylation, and thus for transduction of PERK-arm mediated UPR signaling. On the other hand, Ca^2+^ did not seem to be required for AZC-mediated upregulation of BiP. The Ca^2+^ sensitivities of the different parts of the UPR signal transduction pathways remain to be determined, but it is already noteworthy that the BiP upregulation is insensitive to both the PERK inhibitor and to BAPTA-AM.

Finally, we found that 6 h of pre-treatment with 5–10 mM AZC reduced the amount of Ca^2+^ released from the ER Ca^2+^ store with TG, but did not induce Ca^2+^ release itself (Figure 8 and Figure 9A). These results are in agreement with those of Caspersen et al. who previously demonstrated in PC12 cells that AZC did not induce Ca^2+^ release from the ER by itself although they also claim that the ER is not significantly depleted after pre-treatment for 4 h with 5 mM AZC [61]. However, in the latter study, no quantification of the Ca^2+^ signal was performed, and on the traces shown the agonist-induced Ca^2+^ release is anyway smaller than in control conditions.

We initially hypothesized that the reduction in TG-induced cytosolic Ca^2+^ rise after pre-incubation with AZC may be due to a reduced Ca^2+^ content in the ER and therefore a lower release of Ca^2+^ into the cytosol upon TG application, or due to an increased Ca^2+^ transfer from ER to mitochondria, thereby preventing detection of the Ca^2+^ in the cytosol. However, when measuring Ca^2+^ simultaneously in the ER and the mitochondria, we did neither observe a reduction in ER store content nor an increase in mitochondrial Ca^2+^ transfer in cells pretreated for 6 h with 5 or 10 mM AZC as compared to control cells (Figure 9A,B). We therefore speculate that pre-treatment with AZC affects Ca^2+^-transport systems outside the ER and mitochondrial compartments. For instance, it is possible that PMCA, responsible for exporting Ca^2+^ from the cytosol to the extracellular environment, is affected. We could exclude a modification in PMCA expression levels (Figure 9C) though a change in activity remains possible. A more efficient export of Ca^2+^ from the cytosol by PMCA may lead to an apparent reduction of the amount of cytosolic Ca^2+^ without an actual decrease of the ER Ca^2+^ store content, and thus explain the reduced τ value for the clearance of Ca^2+^ from the cytosol.

Taken together, our results indicate a critical role for Ca^2+^ in the process of eIF2α phosphorylation and LC3-II production upon AZC treatment. AZC however does not directly affect ER Ca^2+^ store content or ER-to-mitochondria Ca^2+^ transfer and the observed effects on intracellular Ca^2+^ handling are likely not related to its effect on UPR and autophagy.

## Abbreviations

ATF	Activating transcription factor
ATG	Autophagy-related
AZC	l-azetidine-2-carboxylic acid
Baf A1	Bafilomycin A1
BAPTA-AM	1,2-bis(*O*-aminophenoxy)ethane-*N*,*N*,*N*′,*N*′-tetraaceticacid tetra(acetoxy-methyl) ester
BiP	Binding immunoglobulin protein
eIF2α	Eukaryotic translation initiation factor 2α
ECL	Enhanced chemiluminescence
EDTA	Ethylene diamine tetraacetic acid
EGTA	Ethylene glycol tetraacetic acid
ER	Endoplasmic reticulum
ERp	Endoplasmic reticulum protein
FBS	Fetal bovine serum
GAPDH	Glyceraldehyde 3-phosphate dehydrogenase
IP_3_R	Inositol trisphosphate receptor
IRE1	Inositol-requiring enzyme 1
LC3	Microtubule-associated protein light chain 3
MCU	Mitochondrial Ca^2+^ uniporter
mTOR	Mechanistic target of rapamycin
PARP	Poly adenosine diphosphate ribose polymerase
PBS	Phosphate-buffered saline
PERK	Protein kinase RNA-like ER kinase
PI	Propidium iodide
PMCA	Plasma membrane Ca^2+^ ATPase
SDS	Sodium dodecyl sulfate
SEM	Standard error of the mean
SERCA	Sarco-/endoplasmic reticulum Ca^2+^ ATPase
STS	Staurosporine
TBS	Tris-buffered saline
TG	Thapsigargin
UPR	Unfolded protein response
XBP	X-box protein
